# Flash Sintered Potassium Sodium Niobate: High-Performance Piezoelectric Ceramics at Low Thermal Budget Processing

**DOI:** 10.3390/ma15196603

**Published:** 2022-09-23

**Authors:** Ricardo Serrazina, Alexander Tkach, Luis Pereira, Ana M. O. R. Senos, Paula M. Vilarinho

**Affiliations:** 1 Department of Materials and Ceramic Engineering, CICECO—Aveiro Institute of Materials, University of Aveiro, 3810-193 Aveiro, Portugal; 2 CENIMAT-I3N, School of Science and Technology, FCT-NOVA, Universidade NOVA de Lisboa, Campus da Caparica, 2829-516 Caparica, Portugal

**Keywords:** electroceramics, ferroelectrics, lead-free piezoelectrics, KNN, alternative sintering, electrical properties, EBSD

## Abstract

Alternative sintering technologies promise to overcome issues associated with conventional ceramic sintering such as high thermal budgets and CO_2_ footprint. The sintering process becomes even more relevant for alkali-based piezoelectric ceramics such as K_0.5_Na_0.5_NbO_3_ (KNN) typically fired above 1100 °C for several hours that induces secondary phase formation and, thereby, degrades their electrical characteristics. Here, an ability of KNN ceramics to be of high performance is successfully demonstrated, using an electric field- and current-assisted Flash sintering technique at 900 °C only. Reported for the first time, Flash sintered KNN ceramics have room-temperature remnant polarization P_r_ = 21 μC/cm^2^ and longitudinal piezoelectric coefficient d_33_ = 117 pC/N, slightly superior to that of conventional ones due to the reduced content of secondary phases. High-performance KNN ceramics Flash sintered at a low-thermal budget have implications for the development of innovative low carbon technologies, electroceramics stakeholders, and piezoelectric energy harvesters.

## 1. Introduction

Motivated by the need to replace lead-based piezoelectrics, alkali niobates have been reported as one of the most promising family of lead-free piezoelectric compounds for applications in sensors, actuators, and energy-harvesting devices [1]. The interest in potassium sodium niobate (K_0.5_Na_0.5_NbO_3_, KNN) resides also in its high tetragonal to cubic transition temperature (T_C_) and longitudinal piezoelectric coefficient, d_33_ [2,3]. Non-textured and non-doped ceramics present, typically, d_33_ of 80–200 pC/N with the tetragonal symmetry phase being stable up to T_C_ of 420 °C [4].

Conventional production of KNN ceramics meets however some experimental limitations [5,6,7,8]. The high temperature and long sintering time induce the volatilization of alkali elements, and thereby the formation of secondary phases with negative effect on both T_C_ and d_33_ [9]. Among possible solutions such as an initial alkali excess, which has to be adjusted to each particular set of preparation conditions, and sintering aids, which have to be also carefully selected, the use of alternative, low-temperature, sintering methods to densify KNN ceramics [6] looks most attractive. Recently, we have used Spark Plasma Sintering and Texturing (SPS/SPT) to produce high-density ceramics at a temperature of 1000 °C for 20 min, with relatively high d_33_ (95–108 pC/N) and moderate T_C_ (370–386 °C) [10]. However, in SPS/SPT, the use of graphite dies and reducing atmospheres implies a post heat treatment for several hours [10], not to mention the limitation in terms of produced shapes [11].

Flash sintering, an electric field- and current-assisted technique [12], has been presented as a possible solution for the sintering of KNN [13,14,15,16], with reduced thermal budget. Ceramic densification has been shown to occur at a lower temperature and in a much shorter period of time, when an electric field is directly applied to a green body [12,13,14]. However, while a large part of the articles on Flash sintering is focused on the sintering process itself and parameters affecting its equilibration, the piezoelectric and ferroelectric properties of Flash sintered KNN have not been disclosed so far. In this work, these key properties required for piezoelectric applications are reported, for the first time, for Flash sintered KNN ceramics and compared with those of conventionally sintered ceramics.

## 2. Materials and Methods

**Figure 1:**
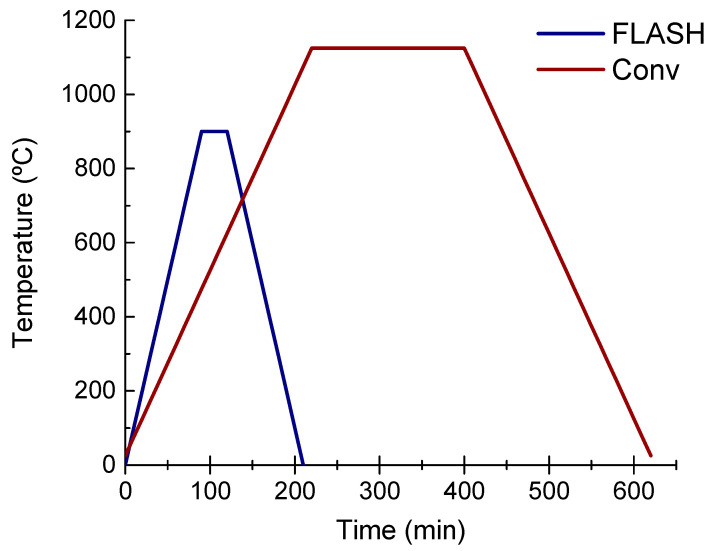
Thermal profiles used for sintering of KNN ceramics by conventional (Conv) (T_max_ = 1125 °C, time_Total_ = 620 min) and Flash (T_max_ = 900 °C, time_Total_ = 210 min) processes. A considerably lower thermal budget is involved for Flash when compared with conventional sintering.

K_0.5_Na_0.5_NbO_3_, KNN, single phase powders were produced by solid-state reaction, using high purity alkali carbonates (K_2_CO_3_, Sigma-Aldrich, St. Louis, MI, USA, 99.99% and Na_2_CO_3_, Sigma-Aldrich, 99.999%) and niobium oxide (Nb_2_O_5_, Alfa Aesar, Haverhill, MA, USA, 99.9%). Powder characterization and processing details are published elsewhere [14,16] as well as Flash sintering setup scheme and mechanism [15]. In short, green compacts (ca. 15 × 5 × 2 mm^3^) were uniaxially and isostatically pressed to a green density of ca. 65%. These pellets were sintered in an adapted contact-dilatometer, with or without the application of an electric field, respectively, for Flash and conventional processes. The conventional sintering (Conv) cycle was optimized to obtain high-density ceramics, using 5 °C/min heating and cooling rates and a sintering temperature of 1125 °C with a 3 h dwell, as shown in Figure 1. Considerably lower thermal budget conditions were used for Flash sintering, as also seen from Figure 1. The pellets were placed in-between two platinum foil electrodes and heated to 900 °C at a rate of 10 °C/min. After an isothermal step of 30 min, a 300 V/cm electric field was applied, and the current limit set to 20 mA/mm^2^, while the holding time after the Flash event was 60 s [14]. A decrease of 20% in the maximum furnace temperature and 66% in the cycle time was accomplished when using Flash instead of the conventional sintering.

The microstructure and local structure of the sintered ceramics were analysed using field-emission scanning electron microscope (SEM, Hitachi SU-70, Tokyo, Japan) equipped with an Electron Backscattered Diffraction (EBSD) detector (EBSD Bruker e-Flash Quantax CrystAlign, Billerica, MA, USA) at 25 kV acceleration potential. EBSD phase analysis is done using ESPRIT software. Prior to the analysis, ceramics were polished using SiC papers, diamond paste and colloidal silica. The density of the ceramics was determined by the Archimedes method with correction for the open porosity on at least three specimens, using water as the immersion liquid.

To access the dielectric, ferroelectric and piezoelectric behaviour of KNN ceramics, 1 mm thick specimens with ca. 5 × 3 mm^2^ section areas were prepared from sintered bodies, using a diamond cutting wire and SiC papers for thickness reduction. After a fine polishing (SiC P2500), platinum electrodes were brush painted (SPI-CHEM 04990-AB, West Chester, PA, USA) at the opposite faces of the ceramics, for a parallel-plate-capacitor configuration. A drying step at 50 °C was employed, followed by a cure and sintering electrode process, according to the manufacturer indications (maximum temperature of 900 °C, for 1 h).

The real part of the relative dielectric permittivity (ε_r_) and dissipation factor (tanδ) were accessed using a precision LCR-meter (HP 4284A, Santa Clara, CA, USA), with a 1 V oscillation potential and the frequency of 1 MHz. The temperature dependence was obtained on cooling the ceramics after heating in a tubular furnace with an alumina sample holder, using a 2 °C/min rate, and a dwell time of 2 min before each measurement. The polarization, as a function of the AC electric field of the sintered ceramics, was assessed at 1 kHz and at room temperature with a ferroelectric analyser (aixACCT, TF Analyzer 2000, Aachen, Germany). The longitudinal piezoelectric coefficient (d_33_) of these KNN ceramics was measured after a non-destructive Corona poling step at 70 °C, for 15 min, under the potential of 10 kV, followed by an additional 15 min step, at 65 °C, under 12.5 kV. A Berlincourt-type piezoelectric meter (Sinocera YE 2730A, Shanghai, China) with force frequency of 110 Hz and amplitude of 0.25 N was used. Several measurements were performed as on the same specimen as on different ceramics to determine an average d_33_ value and the respective standard deviation.

## 3. Results and Discussion

**Figure 2:**
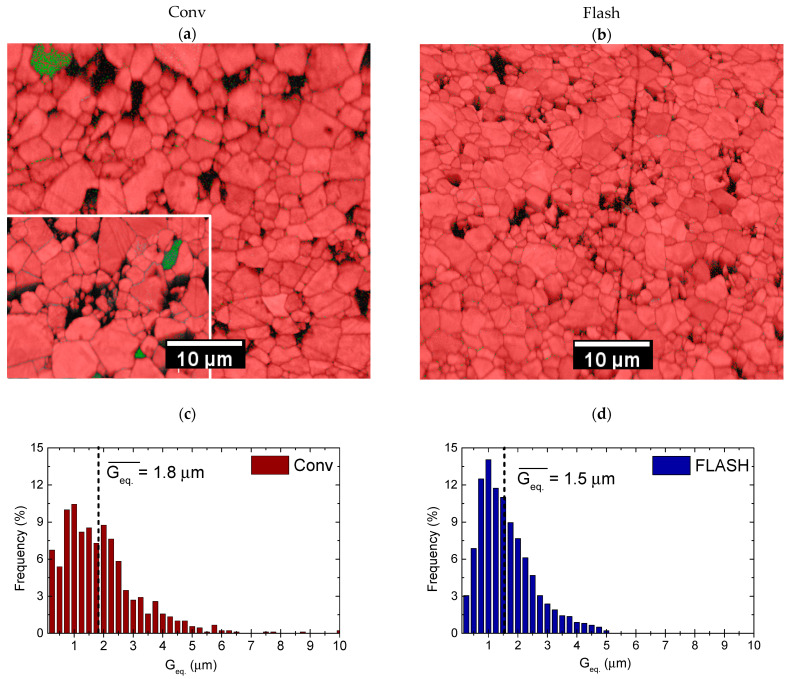
EBSD-phase mapping (**a**,**b**) and equivalent grain size (G_eq._) distribution (**c**,**d**) deduced from them for conventional (**a**,**c**) and Flash (**b**,**d**) sintered KNN ceramics. Red colour in the maps indicates the indexation with JCPDF file 01-085-7128, K_0.5_Na_0.5_NbO_3_ orthorhombic symmetry phase, and green colour with file 04-007-9405, K_0.8_Nb_5_O_15_ tetragonal secondary phase. Inset of (**a**) reveals another EBSD mapping for Conv KNN with the secondary phase being systematically indexed in several grains.

KNN Flash and Conv ceramics have a relative density of 93 ± 3% and 96 ± 2%, respectively. Dense microstructure with an estimated porosity between 3% and 5% for both Conv and Flash KNN ceramics is also seen in SEM-EBSD micrographs, shown in Figure 2a,b, respectively, although some grains were evidently pulled out during polishing that is particularly well seen for Conv KNN with larger grains. The grain size distributions from 0.25 to 10.0 μm and from 0.25 to 5.0 μm are seen in Figure 2c,d for Conv and Flash KNN ceramics, respectively. The average equivalent grain size ($\bar{G_{\mathrm{eq}.}}$) is almost 1.8 μm for Conv KNN and ~1.5 μm for Flash ceramics. Thus, not only the smaller average grain size is obtained by the Flash process, but also the ceramic grain size distribution is narrower, when compared with conventional sintering, as evidenced in Figure 2.

Regarding the local structure, the EBSD phase-maps in Figure 2a,b, reveal grains separated by a dark colour, indicating a discontinuity in the crystal structure, orientation, or composition. The red colour is associated with K_0.5_Na_0.5_NbO_3_ orthorhombic symmetry phase (JCPDF file 01-085-7128), while the green colour corresponds to Nb-rich tetragonal symmetry of K_0.8_Nb_5_O_15_ secondary phase (JCPDF file 04-007-9405). Conv KNN ceramics evidently contains grains of the Nb-rich phase as clearly seen in Figure 2a. The inset of Figure 2a shows that this secondary phase is systematically observable throughout the conventionally sintered ceramics. In contrast, secondary phases are hardly detectable in Flash ceramics, with just a few small indexations at the grain boundaries (see Figure 2b). Several EBSD analyses consistently revealed the concentration of the secondary phase in Conv KNN to be 0.25 to 0.50 vol.%, whereas that for Flash ceramics was found to be <0.05 mol.%.

**Figure 3:**
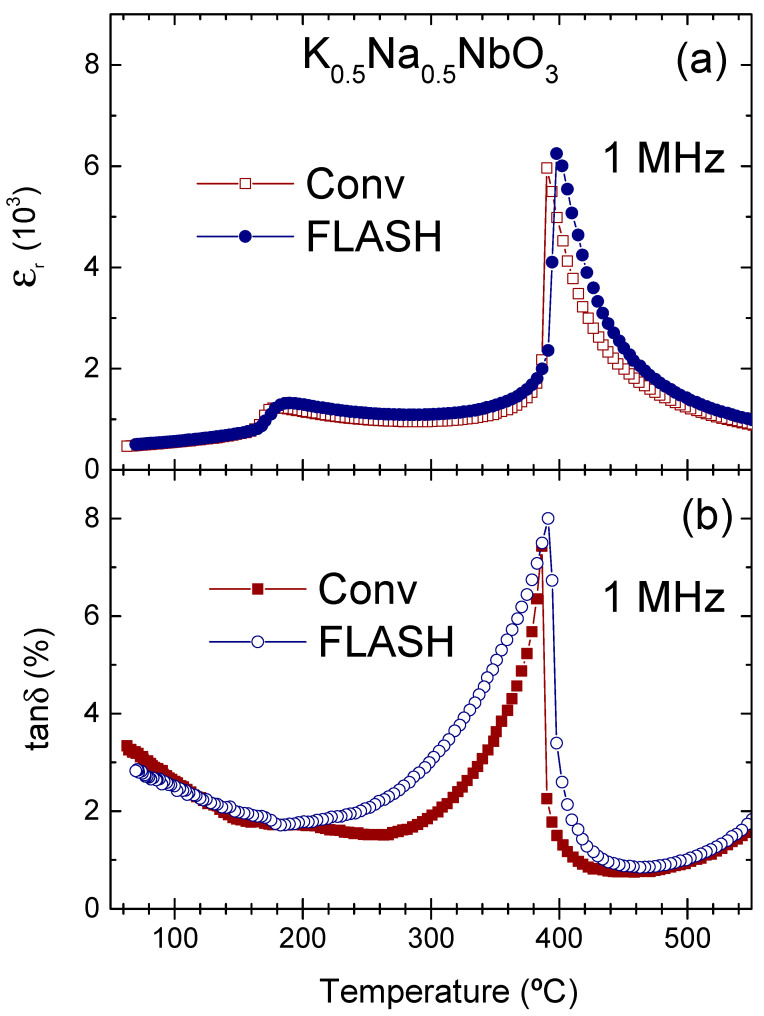
Real part of the relative dielectric permittivity, ε_r_ (**a**), and dissipation factor, tanδ (**b**), of conventionally (squares) and Flash (circles) sintered KNN ceramics measured as a function of temperature, at 1 MHz, on cooling.

The variation of the relative dielectric permittivity and dissipation factor of Conv and Flash KNN ceramics measured on cooling at a frequency of 1 MHz is demonstrated as a function of the temperature in Figure 3a,b, respectively. A pair of peaks in the temperature dependence of the permittivity is observed and associated with structural phase transitions, as expected for KNN [4]. For Conv KNN, the orthorhombic to tetragonal phase transition corresponds to a peak at T_O__−__T_ = 181 °C, with the peak ε_r_ = 1225, and the tetragonal to cubic phase transition corresponds to another peak at T_C_ = 390 °C, with ε_r_ = 5963. In the case of Flash ceramics, both the peak permittivity values and temperatures are slightly superior when compared with Conv KNN. ε_r_ is of 1321 at T_O__−__T_ = 190 °C and of 6249 at T_C_ = 398 °C. The increase of the relative permittivity and transition temperatures for Flash ceramics compared to Conv KNN can be related to the deviation from the stoichiometry associated with the secondary phases, prevalent in Conv KNN [17] and suppressed in Flash ceramics. This behaviour is accompanied by a respective peak in dissipation factor (tanδ) that occurs at T_C_ for both ceramics. The value of tanδ reaches a maximum of about 8% at T_C_, decreasing towards ≈2% at T_O__−__T_, and then slightly increasing during the further cooling.

**Figure 4:**
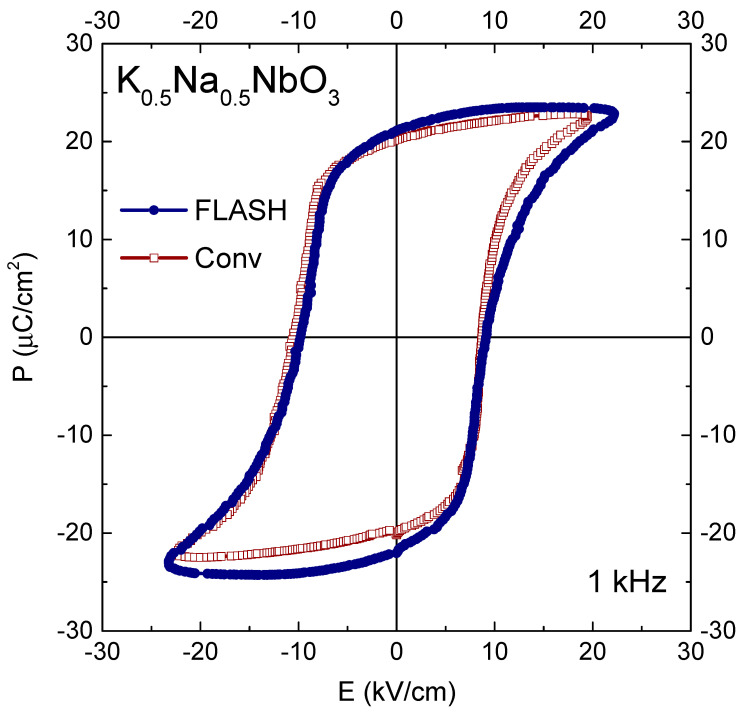
Polarization (P) as a function of applied electric field (E) of conventionally (open squares) and Flash (solid circles) sintered KNN ceramics, measured at 1 kHz and room temperature.

A slight superiority of Flash ceramics over Conv KNN is visible as well in the room-temperature polarization (P) behaviour as a function of the applied AC (1 kHz) electric field (E), illustrated in Figure 4. At a rather similar coercive field of 10 kV/cm, remnant polarization values of 20 and 21 μC/cm^2^ are obtained for Conv and Flash ceramics, respectively. In terms of the piezoelectric performance at room temperature, d_33_ piezoelectric coefficient of 115 for Conv KNN is also slightly lower than 117 pC/N for Flash ceramics.

Table 1 summarizes the most relevant properties of Flash and conventional KNN ceramics studied in this work, presenting also a comparison with other KNN ceramics, produced either by alternative microwave (MW) assisted [18] and SPS/SPT sintering techniques [10] or by conventional processes [18,19], as well as with KNN single crystal [20]. The T_C_ value of 398 °C for Flash KNN is very similar to that reported by Birol and co-workers [19] for conventionally sintered KNN. Furthermore, it is higher than that reported for SPS/SPT ceramics [10]. The decrease in comparison with the T_C_ of 429 °C for KNN single crystal [20] is explained by the presence of impurities, grain boundary-localized secondary phases and crystal structure defects or residual stresses, usually present in ceramics and less common or absent in single crystals. Values of the permittivity and dissipation factor at T_C_ of the produced ceramics are also in agreement with the literature.

A ferroelectric analysis reveals that the remnant polarization for Conv and Flash KNN ceramics is close to those reported for KNN ceramics and single crystals [10,18,19,20]. On the other hand, the coercive field is closer to that of SPT ceramics [10] and KNN single crystals [20], being lower than that for SPS [10] and conventionally sintered KNN reported by Birol et al. [19]. In addition, the piezoelectric coefficient of 117 pC/N determined for Flash ceramics is superior not only compared with Conv KNN of the current work but also with all KNN reported in the literature [10,18,19] except for the [001]-oriented single crystals [20]. Thus, KNN ceramics obtained by Flash sintering in spite of the reduced thermal budget can provide an electrical behaviour similar or in some cases even better than that of ceramics sintered conventionally or by other methods.

## 4. Conclusions

In conclusion, the dielectric, ferroelectric and piezoelectric behaviour of KNN ceramics produced by Flash sintering (T_max_ = 900 °C, time_Total_ = 210 min) was presented for the first time and found to be slightly superior to that obtained using conventional sintering technique (T_max_ = 1125 °C, time_Total_ = 620 min) or by other methods reported in literature. The superior behaviour was explained by the lower content of secondary phase detected using EBSD analysis. Flash sintered KNN ceramics are characterized by ε_r_ = 6249 and tanδ = 0.08 at T_C_ of 398 °C as well as Pr = 21 μC/cm^2^, Ec = 10 kV/cm and d_33_ = 117 pC/N at room temperature. As a result, Flash can be considered as a fast electric field- and current-assisted sintering process with low thermal budget suitable for producing high-performance lead-free piezoelectric ceramics for sensor, actuator, and energy-harvesting applications.

## Figures and Tables

**Table 1:** Relative density (ρ_rel._), average equivalent grain size ($\bar{G_{\mathrm{eq}.}}$), orthorhombic to tetragonal (T_O__−__T_), and tetragonal to cubic (T_C_) transition temperatures, relative dielectric permittivity (ε_r_) and dissipation factor (tanδ) at T_C_ and room temperature (RT), as well as RT remnant polarization (P_r_), coercive field (E_c_) and piezoelectric coefficient (d_33_) of Flash and Conv KNN ceramics of this work in comparison with literature reports.

KNN	ρ_rel._, %	$\bar{G_{eq.}},\;\mu m$	T_O__−__T_, °C	T_C_, °C	ε_r_		tanδ		P_r_, μC/cm^2^	E_c_, kV/cm	d_33_, pC/N	Ref.
					at T_C_	at RT	at T_C_	at RT				
**Flash**	**93 ± 3**	**1.5**	**190**	**398**	**6249**	**527**	**0.080**	**0.043**	**21**	**10**	**117 ± 2**	**This work**
**Conv**	**96 ± 2**	**1.8**	**181**	**390**	**5963**	**468**	**0.074**	**0.034**	**20**	**10**	**115 ± 2**	
Conv	95–96	-	190	400	≈5200	472	-	≈0.04	20	20	110	[19]
Conv	94	6.6	-	401	≈5500	468	≈0.9	0.031	20	12	88	[18]
MW	93.8	3.8	-	398	≈5000	427	≈0.7	0.035	18	12	85	
SPS	96	3.0	207	386	4160	736	0.175	0.377	17	18	95	[10]
SPT	99.8	1.4	204	370	4672	576	0.160	0.045	20	12	108	
Single Crystal	-	-	215	429	29,100	≈300	-	-	19	11	160	[20]

## Data Availability

The data presented in this study are available on request from the corresponding author.
